# N6-methyladenosine-related microRNAs risk model trumps the isocitrate dehydrogenase mutation status as a predictive biomarker for the prognosis and immunotherapy in lower grade gliomas

**DOI:** 10.37349/etat.2022.00100

**Published:** 2022-09-30

**Authors:** Feng Yuan, Yingshuai Wang, Xiangming Cai, Chaonan Du, Junhao Zhu, Chao Tang, Jin Yang, Chiyuan Ma

**Affiliations:** 1 Department of Neurosurgery, Affiliated Jinling Hospital, Medical School of Nanjing University, Nanjing 210002, Jiangsu, China; 2 Department of Internal Medicine III, University Hospital Munich, Ludwig Maximilians-University Munich, 80807 Munich, Germany; 3 School of Medicine, Southeast university, Nanjing 210002, Jiangsu, China; 4 Department of Neurosurgery, Jinling Hospital, The First School of Clinical Medicine, Southern Medical University, Nanjing 210002, Jiangsu, China; 5 Department of Neurosurgery, The Affiliated Jinling Hospital of Nanjing Medical University, Nanjing 210002, Jiangsu, China; Tianjin Medical University General Hospital, China

**Keywords:** Lower grade gliomas, N6-methyladenosine, microRNAs, immune infiltrate, nomogram

## Abstract

**Aim:**

Lower grade gliomas [LGGs; World Health Organization (WHO) grades 2 and 3], owing to the heterogeneity of their clinical behavior, present a therapeutic challenge to neurosurgeons. The aim of this study was to explore the N6-methyladenosine (m^6^A) modification landscape in the LGGs and to develop an m^6^A-related microRNA (miRNA) risk model to provide new perspectives for the treatment and prognostic assessment of LGGs.

**Methods:**

Messenger RNA (mRNA) and miRNA expression data of LGGs were extracted from The Cancer Genome Atlas (TCGA) and Chinese Glioma Genome Atlas (CGGA) databases. An m^6^A-related miRNA risk model was constructed via least absolute shrinkage and selection operator (LASSO), univariate, and multivariate Cox regression analysis. Next, Kaplan-Meier analysis, principal-component analysis (PCA), functional enrichment analysis, immune infiltrate analysis, dynamic nomogram, and drug sensitivity prediction were used to evaluate this risk model.

**Results:**

Firstly, six m^6^A-related miRNAs with independent prognostic value were selected based on clinical information and used to construct a risk model. Subsequently, compared with low-risk group, LGGs in the high-risk group had a higher m^6^A writer and reader scores, but a lower eraser score. Moreover, LGGs in the high-risk group had a significantly worse clinical prognosis than those in the low-risk group. Simultaneously, this risk model outperformed other clinicopathological variables in the prognosis prediction of LGGs. Immune infiltrate analysis revealed that the proportion of M2 macrophages, regulatory T (Treg) cells, and the expression levels of exhausted immune response markers were significantly higher in the high-risk group than in the low-risk group. Finally, this study constructed an easy-to-use and free dynamic nomogram to help clinicians use this risk model to aid in diagnosis and prognosis assessment.

**Conclusions:**

This study developed a m^6^A-related risk model and uncovered two different m^6^A modification landscapes in LGGs. Moreover, this risk model may provide guidance and help in clinical prognosis assessment and immunotherapy response prediction for LGGs.

## Introduction

Gliomas account for approximately 26% of all primary central nervous system (CNS) tumors and 81% of malignant tumors [1]. Lower grade gliomas [LGGs; World Health Organization (WHO) grades 2 and 3] often occur in the adult cerebral hemispheres and exhibit infiltrative growth, with histological types including astrocytomas and oligodendrogliomas [1, 2]. Due to the highly invasive character of the tumor, total surgical excision is very challenging, and the residual tumor will lead to recurrence and malignant progression. Some of these patients will progress to WHO grade 4 glioma and glioblastoma multiforme (GBM) within a few months, while others remain stable for many years. As a result, LGGs have a wide range of survival, from 1 year to 15 years [3]. There is no standard treatment for LGGs, and the choice of treatment varies depending on the extent of resection, histological subtype, WHO grade and molecular detection results, including surgery, chemotherapy and radiotherapy [4].

Although the histological classification of LGGs has been applied until now, it presents intra- and inter-observer heterogeneity and does not adequately predict clinical prognosis. Therefore, clinicians are increasingly depending on molecular genetic classification to guide clinical decision-making [5, 6]. Isocitrate dehydrogenase (IDH) mutations (including mutations in IDH1 or IDH2) characterize more than 70% of adult LGGs and define a subtype associated with a better prognosis [7]. Oligodendrogliomas typically have both IDH mutations and deletions in the 1p and 19q arms of chromosome (1p/19q coding deletion) and are sensitive to radiochemotherapy and have longer survival than LGGs without these alterations [8, 9]. Alpha-thalassemia/mental retardation syndrome X-linked (ATRX) mutation and loss of expression, and tumor protein p53 (TP53) mutation are more frequent in astrocytomas and telomerase reverse transcriptase (TERT) promoter mutation confers favorable prognosis in adult LGGs with IDH1/2 mutations, these are also important markers of clinical behavior [10, 11].

N6-methyladenosine (m^6^A) is the most common post-transcriptional modification in eukaryotic messenger RNA (mRNA) and is also engaged in regulating transfer RNA, ribosomal RNA and non-coding RNA [12, 13]. The m^6^A modification is primarily catalyzed by the methyltransferase (writer proteins), which is removed by the demethylase (eraser proteins) and recognized by the m^6^A binding protein (reader proteins) [14]. An increasing number of studies have shown that m^6^A modifications play an important role in both physiological and pathological processes (circadian rhythm, gene expression, cell differentiation, stress response, immune response and tumorigenesis) [15–20]. With the rapid development of RNA immunoprecipitation (RIP), next-generation sequencing (NGS) technologies, liquid chromatography (LC) and other detection technologies, the research on the mechanism of m^6^A in tumorigenesis and development has made rapid progress, and targeting m^6^A has also become a new direction for tumor clinical treatment [21].

MicroRNAs (miRNAs) are a class of non-coding RNAs with 19–22 nucleotides. Primary miRNAs produced by RNA polymerase II transcription are converted into precursor miRNAs with Drosha enzymes. The subsequent process is completed in the cytoplasm by dicerase and generates mature miRNAs.

miRNAs induce its cleavage by identifying the corresponding complementary sequence in the 3’ untranslated regions (UTRs) [22]. miRNAs have been proven to be dysfunctional in many types of tumors, and there are many mechanisms that lead to dysfunction, including genomic alterations in the genes encoding miRNAs, aberrant transcriptional regulation of miRNAs, and epigenetic alterations. Studies have also confirmed that miRNAs are involved in regulating tumor development, such as cell proliferation, apoptosis, tumor invasion, and angiogenesis [23]. Recent studies have found that they are also involved in the pathogenesis of glioma. Many studies have shown that some miRNAs are correlated with the diagnosis and prognosis of gliomas [24–26].

In this study, the transcriptional profiles of 2,157 miRNAs and 23 m^6^A genes were extracted from The Cancer Genome Atlas (TCGA) database. Then, we used Pearson’s correlation analysis, least absolute shrinkage and selection operator (LASSO) regression analysis, univariate and multivariate Cox regression analysis to ultimately screen six m^6^A-related miRNAs and construct a risk model. The model was a novel prognostic model constructed based on m^6^A modifications. Next, we screened drug candidates with m^6^A-associated miRNA signature through a public drug sensitivity database. Additionally, we estimated the relationship of this risk model with immune microenvironment and immunotherapeutic response. Finally, we developed an easy-to-use dynamic nomogram to predict the clinical prognosis of LGGs.

## Materials and methods

### Patients

The TCGA database was downloaded from UCSC Xena (https://xenabrowser.net/), which included 530 samples of LGG gene expression RNA sequencing (RNAseq) from the IlluminaHiSeq_RNASeqV2. 524 samples of LGG miRNA mature strand expression RNAseq from the IlluminaHiSeq_miRNASeq, relevant clinical information and 511 samples of LGG somatic mutation. The Chinese Glioma Genome Atlas (CGGA) database was downloaded from the CGGA website (http://www.cgga.org.cn/), which included 198 samples of GBMLGG (including 107 LGG and 91 GBM) gene expression miRNA from the V2.0 miRNA Expression BeadChip (Illumina). LGGs with missing overall survival (OS) values were excluded in order to reduce statistical bias during the analysis.

### Selection of m^6^A genes and m^6^A-related miRNAs

We obtained the transcriptional profiles of miRNAs and m^6^A genes from the TCGA database. A total of 23 m^6^A regulators were included for study in this research, including writers (*CBLL1*, *KIAA1429*, *METTL14*, *METTL3*, *RBM15*, *RBM15B*, *WTAP* and *ZC3H13*), erasers (*ALKBH5* and *FTO*), and readers (*ELAVL1*, *FMR1*, *HNRNPA2B1*, *HNRNPC*, *IGF2BP1*, *IGF2BP2*, *IGF2BP3*, *LRPPRC*, *YTHDC1*, *YTHDC2*, *YTHDF1*, *YTHDF2* and *YTHDF3*) [27]. LASSO regression analysis was applied using the R package glmnet, and 13 m^6^A genes were obtained to be significantly correlated with the prognosis of LGGs. Then, we screened m^6^A-related miRNAs by Pearson’s correlation analysis, and 434 m^6^A-related miRNAs were identified. The criteria for screening by this procedure was |Pearson R| > 0.3.

### Immunofluorescence and immunohistochemistry

Immunofluorescence and immunohistochemistry of m^6^A-related regulatory proteins in cerebral cortex and LGGs were obtained from the Human Protein Atlas (HPA) database (http://www.proteinatlas.org/).

### Risk signature construction and validation

The TCGA database was randomly divided into a training dataset and a validation dataset. Baseline characterization showed no statistically significant differences in molecular and clinical characteristics between these two cohorts (*P* > 0.05) (Table 1). Next, using univariate Cox regression analysis, we screened prognosis-related miRNAs from the 434 m^6^A-related miRNAs in the TCGA database (*P* < 0.05). Using the R package glmnet for LASSO regression, we screened 22 m^6^A-related miRNAs that were significantly associated with OS in LGGs. Then, 22 m^6^A-related miRNAs were identified using multivariate Cox regression analysis to identify six m^6^A-related miRNAs with independent prognostic value. Finally, those six m^6^A-related miRNAs were used to construct a risk model. The risk scores were calculated as follows. Risk score = coef(miRNA1) × expr(miRNA1) + coef(miRNA2) × expr(miRNA2) + ...... + coef(miRNAn) × expr(miRNAn), where coef(miRNAn) is the coefficient of prognosis-related miRNAs and expr(miRNAn) is the expression of miRNAs. The classification of low- and high-risk groups was defined by the mean of the risk scores.

### Bioinformatic analysis

The Database for Annotation, Visualization and Integrated Discovery (DAVID; https://david.ncifcrf.gov/) is used to carry out enrichment analysis. ESTIMATE was used to assess the percentage of stromal and immune cells in the tumor. The proportions of various immune cells in the tumor were evaluated using CIBERSORT. The R package maftools and the tumor immune dysfunction and exclusion (TIDE) algorithm were respectively used to analyze mutation data and immunotherapy response prediction. Single sample gene set enrichment analysis (GSVA; ssGSVA) was performed using GSVA to obtain a functional enrichment score for each sample [28].

### Principal-component analysis and Kaplan-Meier survival analysis

High-dimensional data of 23 m^6^A genes and 6 m^6^A-associated miRNAs for risk modeling were efficiently downscaled, model identified and grouped for visualization using principal-component analysis (PCA). Kaplan-Meier survival analysis was used to assess the prognosis between the low- and high-risk groups.

### Exploration of promising clinical therapeutic compounds targeting m^6^A-related miRNA risk model

To identify potential compounds for clinical treatment of LGGs, the half-maximal inhibitory concentration (IC_50_) of listed compounds were obtained from the Genomics of Drug Sensitivity in Cancer (GDSC) website. The R package oncoPredict was used to predict the IC_50_ of the above compounds in patients with LGG.

### Independence of the m^6^A-related miRNA model

Univariate and multivariate Cox regression analyses were performed to identify independent variables for risk model and other clinicopathological characteristics of LGG patients. Simultaneously, we also comprehensively considered whether there is interaction and correlation between variables via R package corrgram and car.

### Nomogram model construction and validation

The predictive performance of the nomogram and other variables [risk score, age, WHO grade, Karnofsky performance status (KPS), and histological type] for the 1-, 3-, and 5-year OS was established. The receiver operating characteristic (ROC) curve is used to evaluate the discrimination ability of the model. A network-based interactive dynamic nomogram application was constructed using Shiny version 1.6.0.

## Results

### Selection of m^6^A-related miRNAs in LGGs

**Figure 1.**
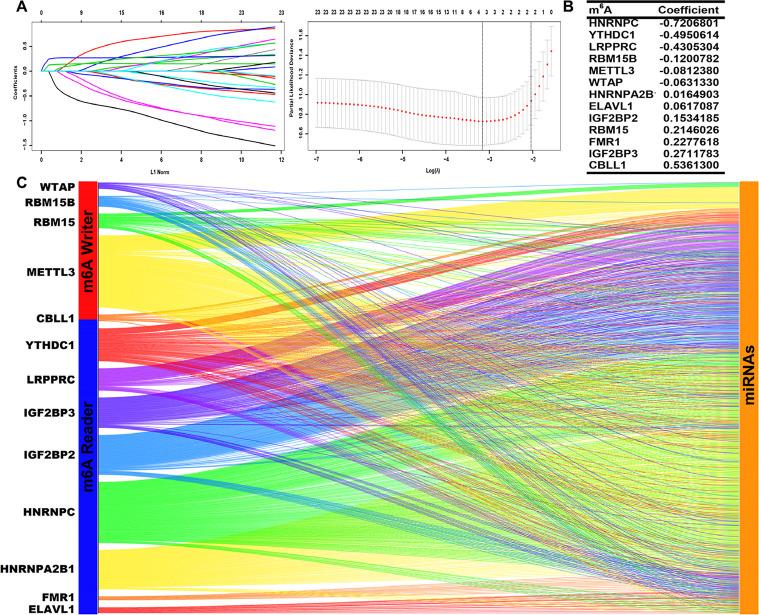
Identification of prognostic m^6^A genes in LGG patients. (A, B) LASSO regression analysis using 10-fold cross-validation; (C) Sankey plots of m^6^A genes and m^6^A-related miRNAs

**Figure 2.**
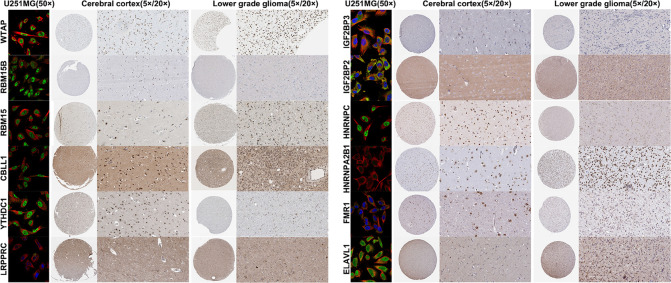
Immunofluorescence and immunohistochemistry of m^6^A-related regulatory proteins in cerebral cortex and low-grade gliomas

We screened prognostic m^6^A genes from 23 m^6^A genes in the TCGA database using LASSO regression analysis (Figure 1A, 1B). We defined m^6^A-related miRNAs as miRNAs associated with one or more of the 13 m^6^A genes (|Pearson R| > 0.3). Sankey plots were used to visualize the m^6^A-miRNA co-expression network (Figure 1C), and 434 m^6^A-related miRNAs were discerned as m^6^A-related miRNAs. Finally, immunofluorescence results revealed that m^6^A-related regulatory proteins were mainly expressed in the nucleus and cytoplasm of glioma cells. Immunohistochemical results demonstrated that WTAP, RBM15, IGF2BP2, HNRNPA2B1, and ELAVL1 were more expressed in LGG than in cerebral cortex, while YTHDC1 and LRPPRC were more expressed in cerebral cortex than in LGG (Figure 2, Figure S1).

### Risk signature construction and validation

**Figure 3.**
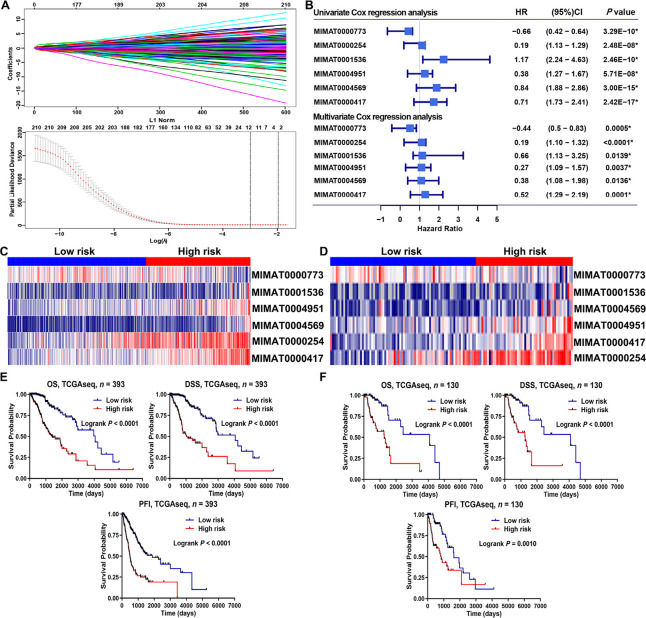
Risk model for LGG patients based on m^6^A-related miRNAs. (A) LASSO regression analysis using 10-fold cross-validation; (B) univariate and multivariate Cox regression analysis showed six independent prognostic miRNAs; (C, D) heat plots displayed the expression of six m^6^A-related miRNAs in the training and validation cohorts; (E, F) Kaplan-Meier analysis of the prognosis in the low-risk and high-risk groups. HR: hazard ratio; CI: confidence interval; ^*^ statistical significance

We screened m^6^A-related prognostic miRNAs from 434 m^6^A-associated miRNAs in the TCGA database using univariate Cox regression analysis. Those m^6^A-related miRNAs that were significantly associated with prognosis were screened in the TCGA database (*P* < 0.05). We further obtained 22 m^6^A-related miRNAs that were significantly associated with OS through the LASSO regression analysis (Figure 3A). Next, in the TCGA database, six m^6^A-related miRNAs with independent prognostic value were selected based on clinical information and used to construct a risk model (Figure 3B). The correlation analysis revealed that the six m^6^A-related miRNAs were significantly correlated with m^6^A regulators (Figure S2A). Then, we found that MIMAT0004951 (MIR887) has m^6^A modification sites (Figure S2B) based on these six miRNA transcripts through online website sequence-based RNA adenosine methylation site predictor (SRAMP; a mammalian m^6^A sites predictor).

To assess the prognostic ability of the risk model, the LGG sample was divided into low- and high-risk groups based on the mean of the risk scores in the training and validation cohorts. The heatmap depicts the expression of six m^6^A-related miRNAs involved in the construction of the risk model in the training cohort (Figure 3C) and the validation cohort (Figure 3D). Kaplan-Meier survival analysis of both the training and validation cohorts showed that patients with LGG in the high-risk group had worse OS than those in the low-risk group (Figure 3E, 3F). To further assess the prognostic predictive ability of the risk model, we included disease-specific survival (DSS) and progression-free interval (PFI) metrics to assess the differences between low- and high-risk LGG patients. The results demonstrated that LGGs in the high-risk group had worse DSS and PFI than low-risk group. The above results suggest that the risk model of m^6^A-related miRNAs can effectively assess the prognosis of LGGs (Figure 3E, 3F). A total of 523 LGG patients, who were included in the study, were randomly divided into a training cohort (*n* = 393) and a validation cohort (*n* = 130). Detailed characteristics for these two cohorts showed homogeneity in these cohorts (Table 1).

**Table 1.** Characteristics of LGGs in the training and validation cohorts

**Characteristics**	**Training dataset**	**Validation dataset**	***P***
Age (year)	42.54 ± 13.66	43.64 ± 12.53	0.3570
KPS	86.51 ± 12.22	86.8 ± 13.87	0.4491
Gender			0.7086
Female	175 (44.5%)	61 (46.9%)	
Male	218 (55.5%)	69 (53.1%)	
Histological_type			0.9071
Astrocytoma	146 (49.7%)	50 (51%)	
Oligodendroglioma	148 (50.3%)	48 (49%)	
WHO_grade			0.2414
WHO grade 2	197 (50.1%)	57 (44.2%)	
WHO grade 3	196 (49.9%)	72 (55.8%)	
Seizure_history			0.6963
Yes	230 (62.2%)	77 (64.7%)	
No	140 (37.8%)	42 (35.3%)	
Sample_type			0.5339
Primary tumor	381 (96.9%)	128 (98.5%)	
Recurrent tumor	12 (3.1%)	2 (1.5%)	
IDH_status			0.1918
Wildtype	75 (19.9%)	18 (14.2%)	
Mutant	302 (80.1%)	109 (85.8%)	
Preoperative_antiseizure			0.7701
Yes	201 (70%)	63 (72.4%)	
No	86 (30%)	24 (27.6%)	
Preoperative_corticosteroids			0.7680
Yes	162 (57%)	53 (59.6%)	
No	122 (43%)	36 (40.4%)	
Headache_history			0.4482
Yes	137 (38.2%)	38 (33.6%)	
No	222 (61.8%)	75 (66.4%)	
Motor_changes			0.8413
Yes	84 (23.9%)	29 (25.4%)	
No	267 (76.1%)	85 (74.6%)	
Sensory_changes			0.4837
Yes	65 (18.5%)	17 (15%)	
No	286 (81.5%)	96 (85%)	

### PCA of m^6^A related miRNA risk signature model

PCA was performed to assess the differences between the low- and high-risk groups based on the transcriptional profiles of 23 m^6^A genes and 6 m^6^A-related miRNAs involved in the construction of the risk model (Figure S2C, 2D). The results of PCA indicated that 6 m^6^A-related miRNAs had better distinction than 23 m^6^A genes in terms of the distribution of the low- and high-risk groups. Next, the ssGSEA results showed compared with low-risk group, LGGs in high-risk group had a higher m^6^A writer and reader scores, but a lower eraser score (Figure S2E).

**Figure 4.**
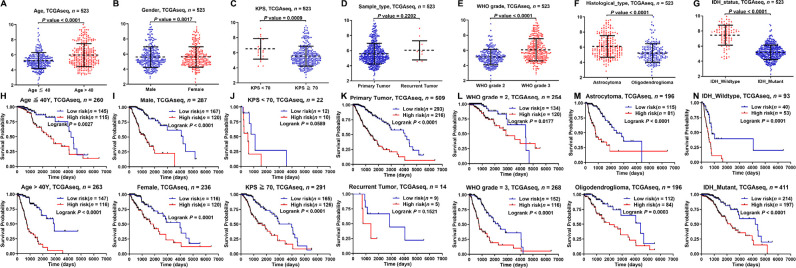
Kaplan-Meier analysis of survival differences stratified by clinicopathological variables between the low- and high-risk groups in the TCGA database. (A–G) Risk score differences stratified by clinicopathological variables between the low- and high-risk groups; (H–N) Kaplan-Meier analysis of survival differences stratified by clinicopathological variables between the low- and high-risk groups

We analyzed the prognostic differences between the low- and high-risk groups in the TCGA and CGGA database stratified by clinicopathological characteristics. First of all, compared with age ≤ 40, KPS ≥ 70, primary tumor, WHO grade 2, oligodendroglioma, and IDH mutant groups, the risk score was higher in age > 40, KPS < 70, recurrent tumor, WHO grade 3, astrocytoma, and IDH wildtype groups (Figure 4A–G; Figure S3A–G). Then, according to the subgroups stratified by age, gender, KPS, sample type, WHO grade, histological type, and IDH status, the OS of the high-risk group continued to be inferior to that of the low-risk group (Figure 4H–N; Figure S3H–N).

### Correlation between m^6^A related miRNA risk signature and immune microenvironment of LGGs

**Figure 5.**
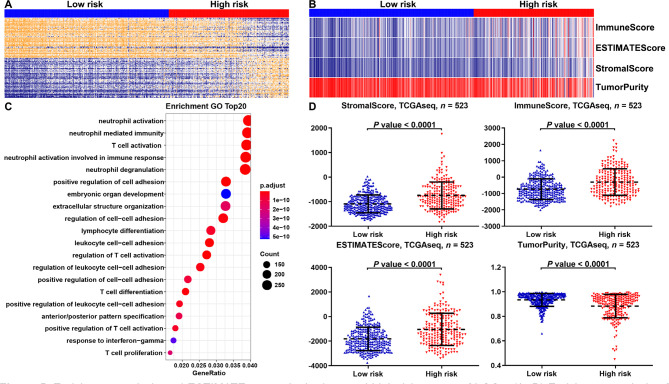
Enrichment analysis and ESTIMATE scores in the low- and high-risk groups of LGGs. (A, B) Enrichment analysis of DEGs in the low- and high-risk groups of LGGs in the TCGA database. The top 20 items were illustrated in the bubblechart; (C) the heat map displayed the distribution features of ESTIMATE scores between the low- and high-risk groups of LGGs in the TCGA database; (D) the scatter plot depicted the results of the ESTIMATE scores between the low- and high-risk groups

We found that the risk model of m^6^A-related miRNAs was mainly correlated with immune-associated biological processes by performing gene ontology (GO) enrichment analysis of differently expressed genes (DEGs) between the low- and high-risk groups (Figure 5A, 5B). Moreover, the ESTIMATE results suggested that compared with low-risk group, LGGs in high-risk group had higher immune and stromal scores, while the purity of tumors was lowered (Figure 5C, 5D).

**Figure 6.**
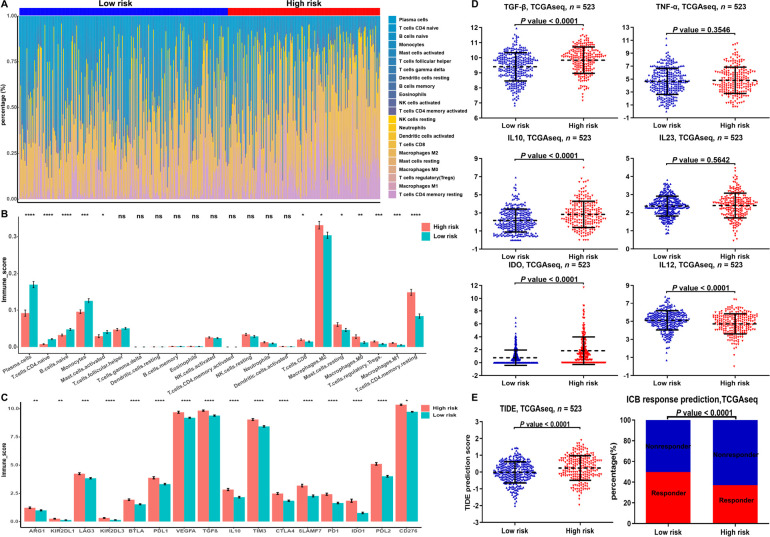
Proportion of TICs and exhausted immune response markers in the low- and high-risk groups of LGGs. (A) Bar chart showed the percentage of various TICs in the samples from the low-risk and high-risk groups; (B, C) bar chart demonstrated the difference in the expression of various immune cells and exhausted immune response markers between the low-risk and high-risk groups; (D) expression of M1- and M2-associated genes differences between the low- and high-risk groups in the TCGA database; (E) TIDE prediction difference in the high- and low-risk patients, and accumulative bar diagram showed that the LGGs with low-risk group (49.67%, 150/302) were more likely to respond to immune checkpoint blockade (ICB) immunotherapy than the LGGs with high-risk group (37.1%, 82/221). ^*^ *P* < 0.05, ^**^ *P* < 0.01, ^***^ *P* < 0.001, ^****^ *P* < 0.0001

We next explored the relationship between risk models of m^6^A-related miRNAs and tumor-infiltrating immune cells (TICs). Immune infiltration analysis revealed higher proportion of M1 macrophage, regulatory T (Treg) cell, M0 macrophage, and M2 macrophage in the high-risk group was than that in the low-risk group (Figure 6A, 6B). We also founded that the expression of exhausted immune response markers was markedly increased in the high-risk group compared to the low-risk group (Figure 6C). Since M1 macrophages exert pro-inflammatory and anti-tumor functions, while M2 macrophages cells have anti-inflammatory and pro-tumor functions, we further analyzed the difference in secretion of immune factors between M1 and M2 macrophages in the low- and high-risk groups. The results showed that the anti-inflammatory cytokines transforming growth factor beta (TGFB), interleukin 10 (IL10) and indoleamine 2,3-dioxygenase (IDO) secreted by M2 macrophages were significantly higher in the high-risk group than in the low-risk group. The pro-inflammatory cytokines tumor necrosis factor (TNF), IL23, and IL12 secreted by M1 macrophages in the high-risk group were not different from those in the low-risk group or were lower than those in the low-risk group (Figure 6D). The above results indicate that M1 macrophages in the high-risk group occurred disability.

In addition, the results of TIDE discovered that LGGs in the low-risk group had a higher response rate to immunotherapy than in the high-risk group. The above results suggest that m^6^A-related miRNA-based risk scores may be a potential predictor of immunotherapy response (Figure 6E).

**Figure 7.**
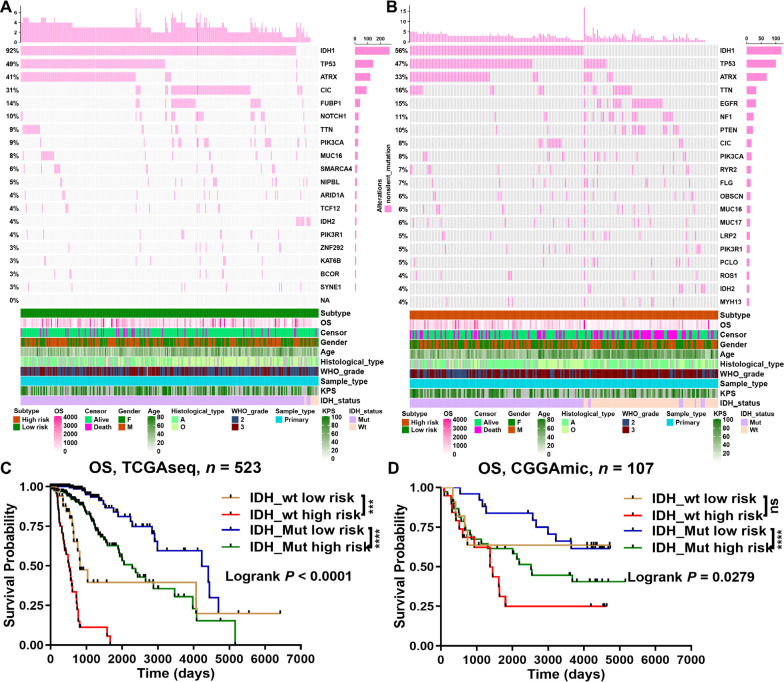
Genetic alteration analyses between the low- and high-risk groups of LGGs. Waterfall plot indicated gene mutations in the (A) low-risk and (B) high-risk groups; (C, D) prognostic evaluation of LGGs survival by IDH mutation status and m^6^A-related miRNA risk score in TCGA and CGGA databases

Then, we analyzed and summarized the mutation data. The waterfall plot shows the top 20 driver genes with the highest frequency of non-silent mutations between the low- and high-risk groups (Figure 7A, 7B). At the same time, the mutation frequency of IDH1 in the low-risk group (92%) was obviously higher than that in the high-risk group (56%). Therefore, we evaluated whether a risk model based on m^6^A-related miRNAs could better predict OS than IDH mutation status. The results showed that LGGs with IDH mutations and IDH wildtype in the high-risk group (defined as IDH_Mut high risk and IDH_wt high risk, respectively) had worse OS than those with IDH mutations and IDH wildtype in the low-risk group (defined as IDH_Mut low risk and IDH_wt low risk, respectively) (Figure 7C, 7D). Interestingly, when OS was less than 2,660 days, LGGs with IDH mutant in the high-risk group (IDH_Mut high risk) had better survival outcomes than those with IDH wildtype in the low-risk group (IDH_wt low risk). However, when OS was greater than 2,869 days (in the TCGA database) or 1,314 days (in the CGGA database), LGGs with IDH mutant in the high-risk group (IDH_Mut high risk) had worse survival outcomes than those with IDH wildtype in the low-risk group (IDH_wt low risk) (Figure 7C, 7D). Therefore, the above results suggest that risk models for m^6^A-related miRNAs are likely to have greater prognostic reference value than IDH mutation status.

### Exploration of promising clinical therapeutic compounds targeting m^6^A-related miRNA risk model

To identify potential drugs for clinical treatment of LGGs, we used the GDSC database to calculate the IC_50_ for each sample using the oncoPredict algorithm. A total of 104 compounds were screened for significant differences in IC_50_ between the low- and high-risk groups. The results of comparing the IC_50_ of the compounds between the two groups revealed that more compounds were more sensitive in the low- risk group compared to the high-risk group (Figure S4A). The difference between the top 5 compounds in the low- and high-risk groups of LGGs (Figure S4B–F).

### Prognostic and clinical characterization of m^6^A-related miRNA risk model

Univariate and multivariate Cox regression analyses revealed risk score, age, WHO grade, and histological type were independent prognostic variables for LGGs (Figure S5A). Simultaneously, the results of the ROC curve analysis indicated that the area under the curve (AUC) of the risk score was always greater than other clinicopathological variables at 1, 3 and 5 years of OS. The above results suggest that the risk model based on six m^6^A-related miRNAs is more reliable for the prognostic evaluation of LGGs (Figure S5B). Moreover, we comprehensively considered whether there is interaction and correlation between variables (Table S1, S2), and found that the risk score and IDH mutation status have a significant correlation (Pearson r = –0.659).

### Construction and evaluation of the prognostic dynamic nomogram

**Figure 8.**
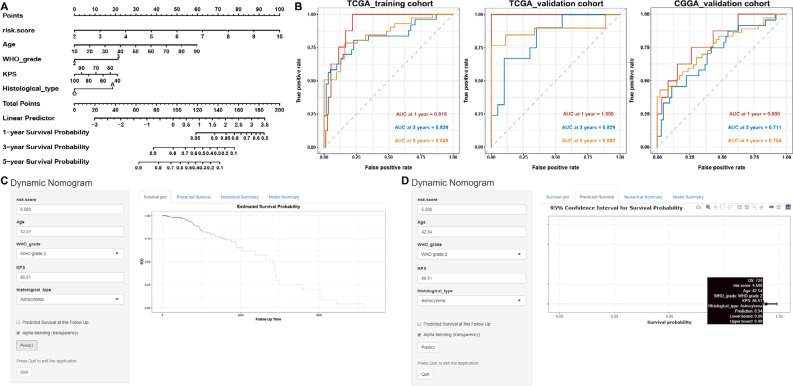
Construction and evaluation of a prognostic nomogram. (A) The nomogram predicts the probability of the 1-, 2-, and 3-year OS; (B) ROC curve of the nomogram predicts the probability of the 1-, 2-, and 3-year OS; (C, D) online dynamic nomogram accessible at https://fengyuan1993.shinyapps.io/LGG_m6A_Immunotherapy/

The nomogram was used to predict the incidence of OS at 1, 3, and 5 years, the risk scores of the nomogram model showed dominant predictive power by comparison with other clinicopathological characteristics (Figure 8A). The ROC curve for the nomogram and its AUC displayed in Figure 8B. The final Cox model incorporated five independent predictors (risk score, age, WHO grade, KPS, and histological type) and was developed as a simple-to-use dynamic nomogram that is available online (https://fengyuan1993.shinyapps.io/LGG_m6A_Immunotherapy/) as screenshotted in Figure 8C, 8D.

## Discussion

Gliomas are the most common primary malignancies of the CNS and are correlated with poor prognosis. The identification of effective diagnostic and prognostic biomarkers is important to guide treatment [29]. An increasing number of studies have confirmed that miRNAs play essential regulatory roles in cells between normal and pathological states [30]. miRNAs have been one of the main focuses of tumor research in the past decade, and many studies have shown the importance of miRNAs in tumor biology, such as promoting tumor growth, invasion, angiogenesis, and immune evasion through the regulation of their target mRNAs [31–33]. In addition, tumor miRNA profiles can define relevant subtypes to predict patient clinical prognosis and guide treatment [34–36]. For example, exosomal miRNAs (miR-210, miR-449 and miR-5194) in plasma of gliomas serve as novel non-invasive biomarkers with high sensitivity to guide the diagnosis and prognosis of patients [37].

m^6^A modification is the most abundant and conserved internal transcriptional modification [18]. It was founded that miRNAs are able to repress gene expression though binding to complementary sequences in the 3’ UTRs of target genes [38]. m^6^A mRNA modification occurs in the region adjacent to the stop codon and close to the proximal region of the 3’ UTRs. In addition, the target sites of miRNAs are abundant in m^6^A peaks. Therefore, the similarity of target sites connected the potential interaction between miRNAs and m^6^A modifications [39]. Previous studies have demonstrated that dysfunction of miRNAs is highly involved in many important biological processes (prenatal development, immune response and tumorigenesis) [40–43]. m^6^A modifications are able to regulate the maturation of those miRNAs that are involved in cell proliferation and tumorigenesis. For example, during pancreatic ductal adenocarcinoma (PDAC) tumorigenesis cigarette smoke condensate (CSC) promotes the maturation of miR-25-3p through mediating METTL3 [44]. METTL3 also enhances the binding of pri-miR-221/222 to DGCR8 to regulate bladder cancer proliferation [45]. m^6^A could mediate arsenate-induced tumorigenesis through modification of several miRNAs (miR-106b, miR-18a/b, miR-3607, miR-423, miR-30a, and miR-320b/d/e) [46]. METTL14, another m^6^A regulator, is shown to suppress the invasion and metastasis of hepatocellular carcinoma (HCC) through promoting the maturation of pri-miR-126 [47].

The role of m^6^A modifications in the malignant progression of gliomas has been increasingly uncovered in recent years. Compared to IDH-mutant gliomas, IDH-wildtype gliomas have a significantly poorer prognosis. The fifth edition of the WHO Classification of Tumors of the CNS for GBM included only the IDH-wildtype [2]. Chang et al. [48] found that METLL3 was significantly overexpressed in IDH-wildtype GBMs, and METLL3 enhanced the stability of long non-coding RNA (lncRNA) metastasis-associated lung adenocarcinoma transcript 1 (MALAT1) through m^6^A modification with the help of human antigen R (HuR) and activated nuclear factor-κB (NF-κB) pathway to promote malignant progression of IDH-wildtype gliomas. Moreover, Chai et al. [49] discovered that YTHDF2 was capable of activating the NF-κB pathway in a manner that facilitated the METTL3-dependent degradation of UBX domain protein 1 (UBXN1) mRNA. Dong et al. [50] also found that under hypoxic conditions, the m^6^A demethylase ALKBH5 was able to promote the formation of an immunosuppressive microenvironment by promoting lncRNA NEAT1-mediated paraspeckle assembly to upregulate C-X-C motif chemokine ligand 8 (CXCL8)/IL8 expression. Both m^6^A and miRNAs are important regulators in the process of tumor development. However, researches on the potential mechanisms and prognostic markers of m^6^A-related miRNAs in LGGs are still absent. In the present study, inspired by the essential biological functions of m^6^A and miRNAs in malignancies, we aimed to establish a risk model based on m^6^A-related miRNAs.

In this study, 434 m^6^A-associated miRNAs were identified from the TCGA database, and then six m^6^A-related miRNAs with independent prognostic value were screened based on clinical information and were used to construct a risk model for m^6^A-related miRNAs to predict the prognosis of LGGs. In particular, miR-346 is shown to inhibit glioma proliferation by targeting nuclear factor I B (NFIB) and may become a potential prognostic and therapeutic candidate [51]. miR-10b is a miRNA that is not expressed in normal brain but is significantly upregulated in glioma. Targeted inhibition of miR-10b significantly suppresses tumor growth in animal models of glioma [52]. The regulation of big mitogen-activated protein kinase 1 (BMK1) by miR-429 has an essential role in glioma invasion [53]. The expression of miR-21, miR-222 and miR-124-3p in serum exosomes was significantly higher in high-grade gliomas than in LGGs and normal controls, and the expression levels decreased rapidly after surgery. This finding implied that exosomal miR-21, miR-222 and miR-124-3p provide a minimally invasive assay to guide the clinical management of gliomas [54]. miRNAs in cerebrospinal fluid (CSF) also have the potential to serve as novel biomarkers for detection in patients with glioma. Analysis of CSF from gliomas and patients with various neurological diseases revealed that miR-15b expression levels could effectively differentiate gliomas from non-glioma patients [55]. Combined with the above studies and based on the transcripts of these six miRNAs, we discovered that only miR-887 had a potential m^6^A modification site through the online website SRAMP. The other five miRNAs were not predicted to contain potential m^6^A modification sites, but their m^6^A modification status should be clarified by experiments such as methylated RIP owing to their essential roles in several types of tumors, including glioma. In addition, miR-887 was first proposed by our team in glioma. We will explore the characteristics of its role in GBM and its relationship with m^6^A modification in future studies.

Subsequently, univariate and multivariate Cox regression analyses revealed a risk model for m^6^A-related miRNA as an independent prognostic risk factor for LGGs. ROC analysis indicated that this risk model outperformed other clinicopathological characteristics in the prognosis prediction of LGGs. We also developed a dynamic nomogram, which showed a highly consistent between observed and predicted rates for 1-, 3-, and 5-year OS. The risk model based on six m^6^A-related miRNAs is highly accurate and the predictive model will be mined for new biomarkers for subsequent studies.

The TIDE prediction score, an algorithm developed for predicting immunotherapy, has been adopted in an increasing number of studies and its predictive ability has been successfully validated [56]. In our study, the results of the TIDE algorithm analysis showed that patients in the low-risk group had a significantly better response rate to immunotherapy than the high-risk group. Immune infiltration analysis revealed higher proportion of M1 macrophage, Treg cell, M0 macrophage, and M2 macrophage in the high-risk group was than that in the low-risk group. Since M1 macrophages exert pro-inflammatory and anti-tumor functions, while M2 macrophages cells have anti-inflammatory and pro-tumor functions, we further analyzed the difference in secretion of immune factors between M1 and M2 macrophages in the low- and high-risk groups. The results showed that the immune factors TGFB, IL10, and IDO secreted by M2 macrophages in the high-risk group were obviously higher than that of the low-risk group. The immune factors TNF, IL23, and IL12 secreted by M1 macrophages in the high-risk group were not different from those in the low-risk group or were lower than those in the low-risk group. The above results indicate that M1 macrophages in the high-risk group occurred disability. We also founded that the expression of exhausted immune response markers was markedly increased in the high-risk group compared to the low-risk group. The above results suggest that this prediction model is highly promising to provide a reliable biomarker for tumor immunotherapy. Furthermore, this study provides novel perspectives on the molecular biological mechanisms of m^6^A-related miRNAs in LGGs.

Clinically, IDH mutation status, WHO grade and histological type are important factors influencing the prognosis of LGGs [57]. However, LGGs with the same type often present with different clinical prognosis, suggesting that current classification systems do not provide reliable prognostic predictions and accurately reflect the heterogeneity of LGG [58]. Therefore, exploring potential predictive and therapeutic biomarkers has become an important direction for LGG. The development of m^6^A-related miRNA risk models provides a new approach for prognosis and immunotherapy prediction of LGGs. These results also provide insights into the process and mechanism of m^6^A-modified miRNAs for future studies. In our study, we are also aware of some weaknesses and limitations of the present study. Such as the lack of other clinical databases for external validation and the biological mechanisms of m^6^A-related miRNAs have not been fully elucidated. Therefore, we will collect more LGG samples in our future work to validate the accuracy of this model and explore the functional mechanisms of miRNAs and their relationship with m^6^A modifications.

In conclusions, our study provides an important guidance for the prognostic assessment of LGGs and may help to shed light on the mechanisms of m^6^A-regulated miRNAs. Additionally, the predictive model showed sensitivity in identifying LGGs who may respond well to immunotherapy.

## Abbreviations

CGGA: Chinese Glioma Genome Atlas
CNS: central nervous system
DSS: disease-specific survival
GBM: glioblastoma multiforme
IC_50_: half-maximal inhibitory concentration
IDH: isocitrate dehydrogenase
IL10: interleukin 10
KPS: Karnofsky performance status
LASSO: least absolute shrinkage and selection operator
m^6^A: N6-methyladenosine
miRNA: microRNA
mRNA: messenger RNA
OS: overall survival
PCA: principal-component analysis
PFI: progression-free interval
ROC: receiver operating characteristic
TCGA: The Cancer Genome Atlas
TICs: tumor-infiltrating immune cells
TIDE: tumor immune dysfunction and exclusion
TNF: tumor necrosis factor
UTRs: untranslated regions
WHO: World Health Organization

## Supplementary materials

The supplementary material for this article is available at: https://www.explorationpub.com/uploads/Article/file/1002100_sup_1.pdf.

## Declarations

### Copyright

© The Author(s) 2022.
