# Supplementary material for: N6-methyladenosine-related microRNAs risk model trumps the isocitrate dehydrogenase mutation status as a predictive biomarker for the prognosis and immunotherapy in lower grade gliomas
Source: Explor Target Antitumor Ther. 2022 Sep 30;3(5):553–69. doi: 10.37349/etat.2022.00100 (PMC9549064; doi:10.37349/etat.2022.00100)
Supplement: Supplementary file 1 [file etat-03-1002100-s001.pdf]

A

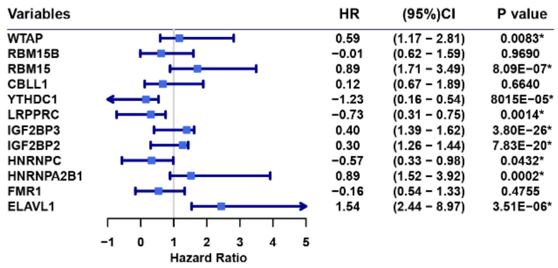

B

m6A_genes	Location(U251MG)	Cerebral cortex				Low grade glioma			
		Gender/Age	Antibody staining	Intensity	Quantity	Gender/Age	Antibody staining	Intensity	Quantity
WTAP	Nuclear	Female/54	Medium	Moderate	75%-25%	Female/37	High	Strong	>75%
RBM15B	Nucleoplasm	Female/64	Low	Weak	<25%	Male/65	Low	Weak	75%-25%
RBM15	Nucleoplasm	Female/19	Low	Weak	<25%	Female/37	High	Strong	>75%
CBLL1	Nuclear	Female/30	High	Strong	>75%	Male/38	Medium	Moderate	>75%
YTHDC1	Nucleoplasm	Female/30	High	Strong	>75%	Female/22	Medium	Moderate	75%-25%
LRPPRC	Mitochondria	Male/57	Medium	Moderate	75%-25%	Male/65	Low	Moderate	<25%
IGF2BP3	Cytosol	Female/52	Not detected	Negative	None	Male/67	Not detected	Negative	None
IGF2BP2	Cytosol	Male/6	Low	Weak	<25%	Male/65	Medium	Moderate	75%-25%
HNRNPC	Nucleoplasm	Male/6	High	Strong	>75%	Male/58	Medium	Moderate	>75%
HNRNPA2B1	Nucleoplasm	Female/45	Medium	Moderate	75%-25%	Male/42	High	Strong	>75%
FMR1	Cytosol	Female/52	Medium	Moderate	75%-25%	Female/22	Medium	Moderate	>75%
ELAVL1	Nucleoplasm,Cytosol	Female/52	Low	Weak	<25%	Female/58	Medium	Moderate	>75%

Figure S1. Immunofluorescence and immunohistochemistry of m^{6}A-related regulatory proteins in cerebral cortex and lower grade gliomas

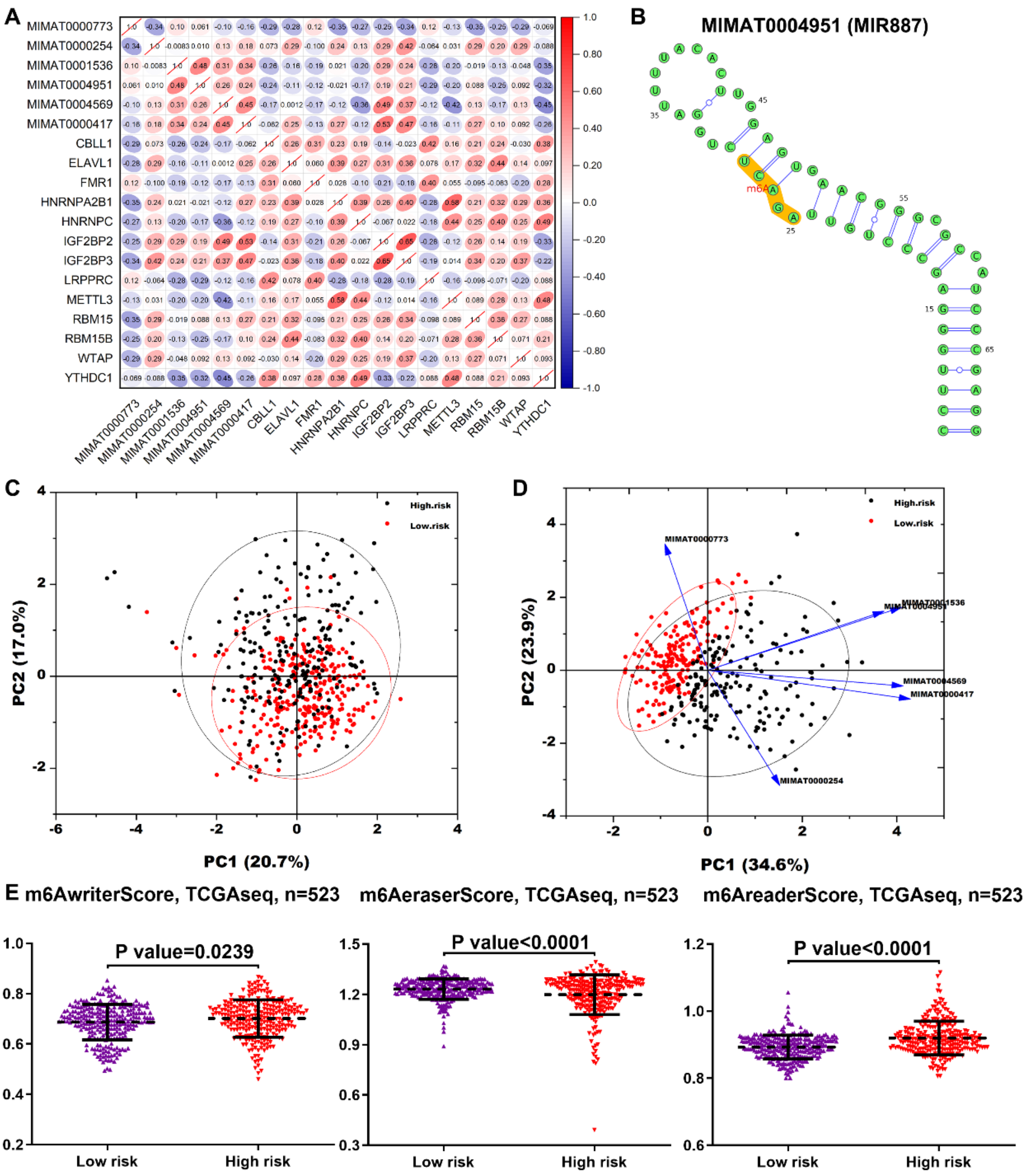

Figure S2. (A) The correlation diagram shows the correlation between six m6A-related miRNAs and m6A regulators. (B) Based on the online website SRAMP for prediction of m6A modification sites in MIMAT0004951 (MIR887). Principal component analysis between the low- and high-risk groups based on (C) 23 m6A genes, and (D) risk model based on the transcriptional profiles of the six m6A-related miRNAs in the TCGA database. (E) The scatter plot showed the ratio differentiation of m6A scores between the low- and high-risk groups

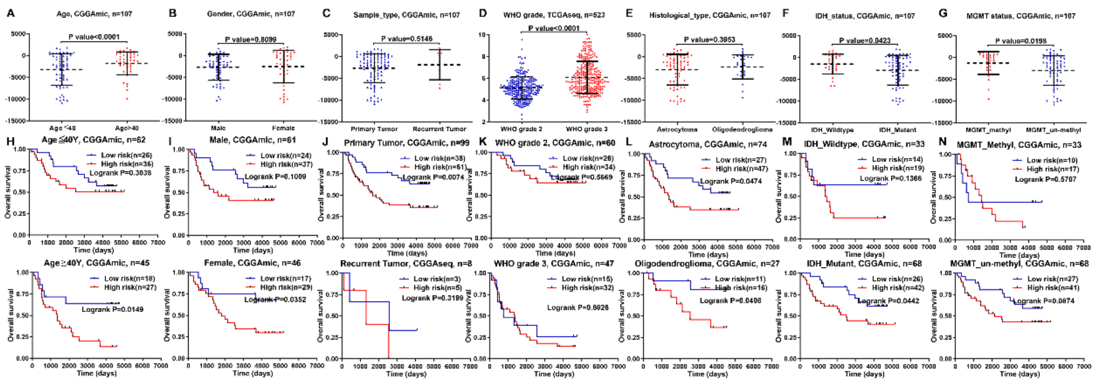

Figure S3. Kaplan-Meier curves of OS differences stratified by age, gender, sample type, WHO grade, histological type, IDH status, and MGMT status between the low- and high-risk groups in the CGGA database. (A–G) Risk score differences stratified by age, gender, sample type, WHO grade, histological type, IDH status, and MGMT status between the low- and high-risk groups in the CGGA database. (H–N) Kaplan-Meier curves of OS differences stratified by age, gender, sample type, WHO grade, histological type, IDH status, and MGMT status between the low- and high-risk groups in the CGGA database

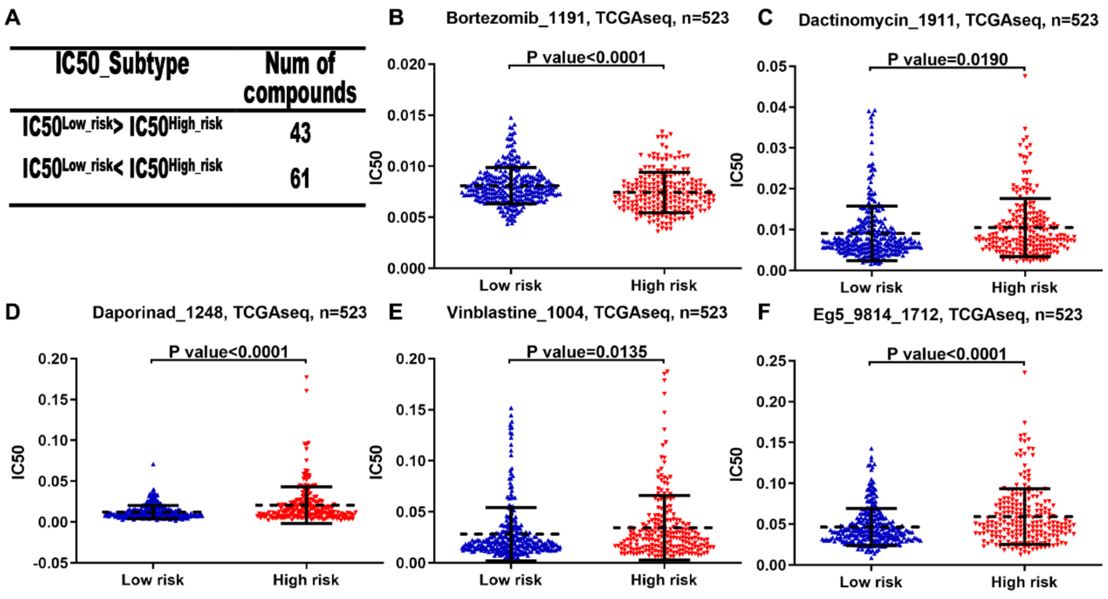

Figure S4. Identification of novel candidate compounds targeting the m6A-related miRNA model. (A) A total of 104 compounds were screened for significant differences between the low- and high-risk groups. (B–F) The difference between the top 5 compounds in the low- and high-risk groups of LGGs

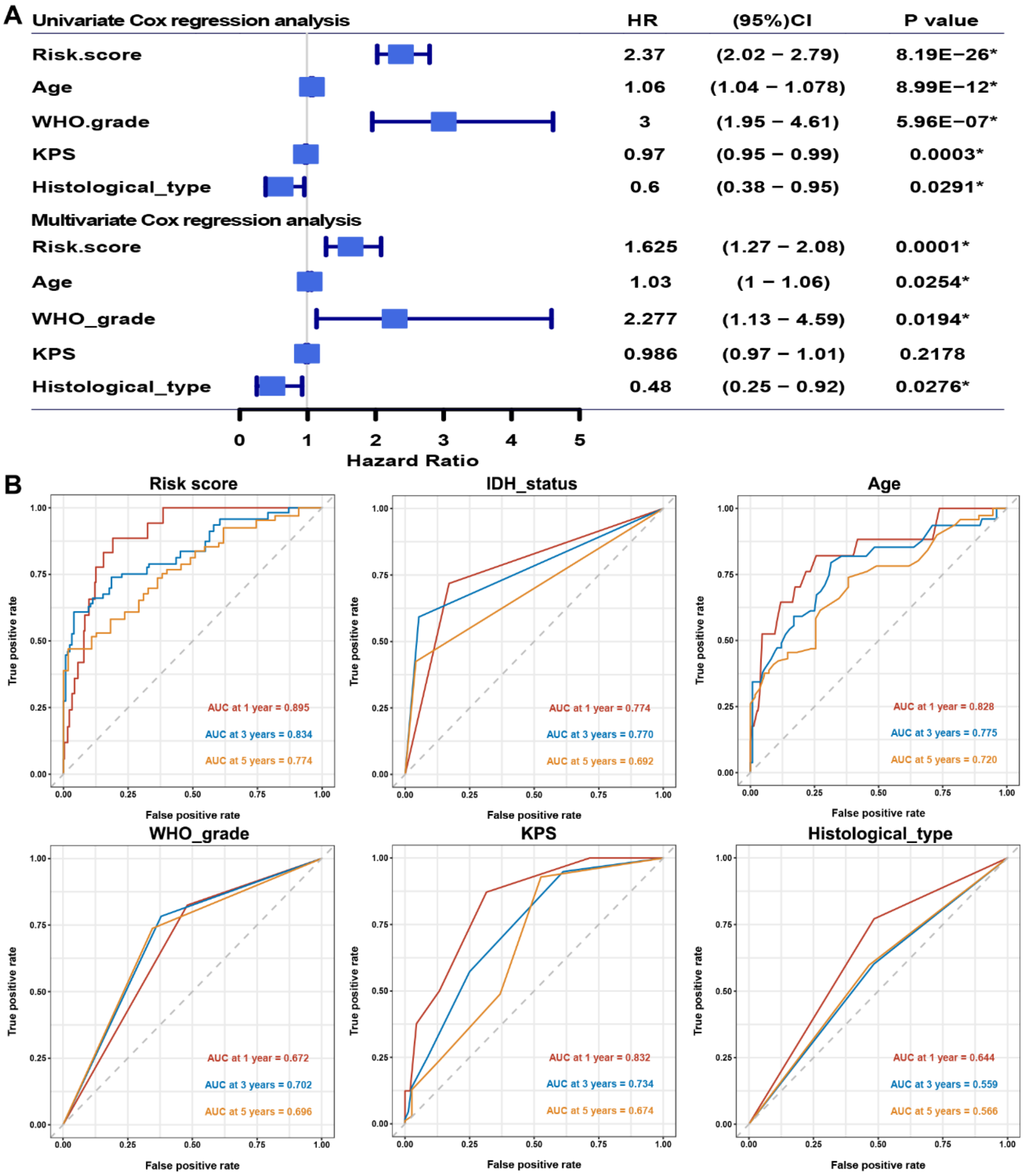

Figure S5. Assessment of the prognostic risk model of the m6A-related miRNAs and clinicopathological characteristics in LGG in the TCGA database. (A) Univariate and multivariate analyses of the clinicopathological characteristics and risk score with the OS. (B) ROC curves of the clinical characteristics and risk score. ROC, receiver operating characteristic. AUC, area under the curve

	risk.score	Gender	Age	Histological	WHO	KPS	IDH_status
**risk.score**	NA	0.1269	0.0022	0.9077	0.4844	0.7661	0.7097
**Gender**	NA	NA	0.7603	0.0563	0.9505	0.6807	0.0017
**Age**	NA	NA	NA	0.3356	0.0496	0.0088	0.0025
**Histological**	NA	NA	NA	NA	0.2491	0.2396	0.4852
**WHO**	NA	NA	NA	NA	NA	0.2122	0.7251
**KPS**	NA	NA	NA	NA	NA	NA	0.1613
**IDH_status**	NA	NA	NA	NA	NA	NA	NA

Table S1. Evaluate whether there is a correlation between the risk model of m6A-related miRNA and other clinicopathological characteristics of LGGs

		risk.score	Gender	Age	Histological	WHO	KPS	IDH_status
**risk.score**	Pearson correlation coefficient	1	0.041	.320**	-.304**	.343**	-.249**	-.659**
	Sig.（2-tailed）		0.423	0	0	0	0	0
	Num	393	393	393	294	393	238	377
**Gender**	Pearson correlation coefficient	0.041	1	-0.003	-0.021	-0.01	-0.022	0
	Sig.	0.423		0.94	0.717	0.84	0.73	0.995

	(2-tailed)			7		9	5	
	Num	393	393	393	294	393	238	377
**Age**	Pearson correlation coefficient	.320**	-0.003	1	.122*	.226**	-.261**	-.320**
	Sig. (2-tailed)	0	0.947		0.036	0	0	0
	Num	393	393	393	294	393	238	377
**Histological**	Pearson correlation coefficient	-.304**	-0.021	.122*	1	-.252**	-0.046	.245**
	Sig. (2-tailed)	0	0.717	0.036		0	0.543	0
	Num	294	294	294	294	294	180	281
**WHO**	Pearson correlation coefficient	.343**	-0.01	.226**	-.252**	1	-0.091	-.250**
	Sig. (2-tailed)	0	0.849	0	0		0.163	0
	Num	393	393	393	294	393	238	377
**KPS**	Pearson correlation	-.249**	-0.022	-.261**	-0.046	-0.091	1	.250**

	coefficient							
	Sig. （2-tailed）	0	0.735	0	0.543	0.163		0
	Num	238	238	238	180	238	238	226
**IDH_status**	Pearson correlation coefficient	-.659**	0	-.320**	.245**	-.250**	.250**	1
	Sig. （2-tailed）	0	0.995	0	0	0	0	
	Num	377	377	377	281	377	226	377

Table S2. Evaluation of the risk model of m6A-related miRNAs and other clinicopathological characteristics of LGGs using multivariate Cox regression analysis. ** At the 0.01 level (2-tailed), the correlation is significant. * At the 0.05 level (2-tailed), the correlation is significant. c Since at least one variable is a constant, it cannot be calculated
